# Effectiveness of Mechanisms and Models of Coordination between Organizations, Agencies and Bodies Providing or Financing Health Services in Humanitarian Crises: A Systematic Review

**DOI:** 10.1371/journal.pone.0137159

**Published:** 2015-09-02

**Authors:** Elie A. Akl, Fadi El-Jardali, Lama Bou Karroum, Jamale El-Eid, Hneine Brax, Chaza Akik, Mona Osman, Ghayda Hassan, Mira Itani, Aida Farha, Kevin Pottie, Sandy Oliver

**Affiliations:** 1 Department of Internal Medicine, American University of Beirut, Beirut, Lebanon; 2 Department of Clinical Epidemiology and Biostatistics, McMaster University, Hamilton, Ontario, Canada; 3 Department of Health Management and Policy, American University of Beirut, Beirut, Lebanon; 4 McMaster Health Forum, McMaster University, Hamilton, Ontario, Canada; 5 Research, Advocacy and Public Policy-making Program, Issam Fares Institute for Public Policy and International Affairs, American University of Beirut, Beirut, Lebanon; 6 VP of Medical Affairs, American University of Beirut, Beirut, Lebanon; 7 Faculty of Medicine, Université Saint Joseph, Beirut, Lebanon; 8 Department of Population Health, London School of Hygiene and Tropical Medicine, London, United Kingdom; 9 Department of Family Medicine, American University of Beirut, Beirut, Lebanon; 10 Department of Psychology, University of Québec, Montreal, Québec, Canada; 11 Faculty of Medicine, American University of Beirut, Beirut, Lebanon; 12 Saab Medical Library, American University of Beirut, Beirut, Lebanon; 13 Department of Epidemiology and Community Medicine, Faculty of Medicine, University of Ottawa, Ottawa, Ontario, Canada; 14 Department of Family Medicine, University of Ottawa, Ottawa, Ontario, Canada; 15 Department of Childhood, Families and Health, Social Science Research Unit, Institute of Education, University of London, London, United Kingdom; University of Geneva, SWITZERLAND

## Abstract

**Background:**

Effective coordination between organizations, agencies and bodies providing or financing health services in humanitarian crises is required to ensure efficiency of services, avoid duplication, and improve equity. The objective of this review was to assess how, during and after humanitarian crises, different mechanisms and models of coordination between organizations, agencies and bodies providing or financing health services compare in terms of access to health services and health outcomes.

**Methods:**

We registered a protocol for this review in PROSPERO International prospective register of systematic reviews under number PROSPERO2014:CRD42014009267. Eligible studies included randomized and nonrandomized designs, process evaluations and qualitative methods. We electronically searched Medline, PubMed, EMBASE, Cochrane Central Register of Controlled Trials, CINAHL, PsycINFO, and the WHO Global Health Library and websites of relevant organizations. We followed standard systematic review methodology for the selection, data abstraction, and risk of bias assessment. We assessed the quality of evidence using the GRADE approach.

**Results:**

Of 14,309 identified citations from databases and organizations' websites, we identified four eligible studies. Two studies used mixed-methods, one used quantitative methods, and one used qualitative methods. The available evidence suggests that information coordination between bodies providing health services in humanitarian crises settings may be effective in improving health systems inputs. There is additional evidence suggesting that management/directive coordination such as the cluster model may improve health system inputs in addition to access to health services. None of the included studies assessed coordination through common representation and framework coordination. The evidence was judged to be of very low quality.

**Conclusion:**

This systematic review provides evidence of possible effectiveness of information coordination and management/directive coordination between organizations, agencies and bodies providing or financing health services in humanitarian crises. Our findings can inform the research agenda and highlight the need for improving conduct and reporting of research in this field.

## Background

Over the past years, man-made and natural disasters have affected large numbers of people worldwide. Considering refugees as an illustrative example, there were 10.4 million refugees and 28.8 million internally displaced people (IDP) worldwide at the beginning of 2013 [1, 2]. The Middle East and North Africa (MENA) region is currently witnessing the largest increase in the number of displaced people mainly due to the armed conflict in Syria. More than 2.5 million Syrian refugees are distributed across Lebanon, Iraq, Jordan, Turkey and Egypt. Lebanon is hosting the largest number, with 1,173,617 Syrian refugees registered in Lebanon as of October 2014 [3]. Populations affected by displacement across and within international borders face high morbidity and mortality [4].

A number of local and international non-governmental organizations (NGOs), United Nations (UN) agencies and governmental bodies and agencies provide humanitarian, including medical and health assistance to displaced people. However, the limited coordination between these organizations and agencies can lead to inefficiencies, duplication in service delivery, and inequity. Geographic inequalities can occur as a result of lack of coordination through the targeting of assistance to favored areas and populations. Confusion may also be caused by differences in donor policies and preferences [5, 6].

The SPHERE project, which aims to improve the quality of the actions of humanitarian NGOs during disaster response, stresses the principle of coordination [7]. Coordination is crucial in humanitarian emergencies. Improved coordination among organizations providing humanitarian aid can enhance the flow of resources and increase the accountability, the effectiveness and the impact of relief efforts [8].

The UN General Assembly resolution 46/182 set the basis of the current international humanitarian coordination system in December 1991. In the Humanitarian Reform of 2005, new elements to improve capacity, predictability, accountability, leadership and partnership were introduced. The creation of the Cluster Approach was the most visible aspect of the reform. Clusters are groups of humanitarian organizations working in the main sectors of humanitarian assistance, e.g. shelter and health, when there are humanitarian needs within a sector and when numerous actors within sectors and national authorities need coordination support. Clusters create partnerships between actors working in providing humanitarian assistances such as international humanitarian organizations, national and local authorities, and civil society [9].

A recent priority-setting exercise by the “Evidence Aid Priority Setting Group” identified the coordination of humanitarian interventions among the top ten priorities for systematic reviews in the area of planning for or response to disasters, humanitarian crises and other major healthcare emergencies [10]. Similarly, the Center for Systematic Review for Health Policy and Systems Research (SPARK) at the American University of Beirut in Lebanon, held in January 2014 a priority setting exercise addressing this issue in the specific setting of refugee health. The discussions suggested that the limited coordination between organizations and agencies delivering health services to refugees is the main problem hindering their work and leading to duplication and inefficiency in the delivery of those services. The stakeholders participating in the meeting were actively engaged in framing and specifying the objective of this review [11].

## Objective

The objective of this review was to assess how, during and after humanitarian crises, different mechanisms and models of coordination between organizations, agencies (UN and others) and governmental bodies providing or financing health services compare in terms of access to health services and health outcomes.

## Methods

### Protocol and registration

We registered a protocol for this review in the PROSPERO prospective register of systematic reviews under registration number PROSPERO 2014:CRD42014009267 and available from http://www.crd.york.ac.uk/PROSPERO_REBRANDING/display_record.asp?ID=CRD42014009267.

### Definition and classification of coordination

We used the following definition of coordination in humanitarian crises: “the systematic use of policy instruments to deliver humanitarian assistance in a cohesive and effective manner. Such instruments include strategic planning, gathering data and managing information, mobilizing resources and ensuring accountability, orchestrating a functional division of labor, negotiating and maintaining a serviceable framework with host political authorities and providing leadership” [12]. We used the classification of coordination proposed by the Joint Evaluation of Emergency Assistance to Rwanda, which consists of four broad categories: information coordination, coordination through common representation (for example, for negotiating access, briefing the media, negotiating funding), framework coordination (requiring a shared sense of priorities) and management/directive coordination [13].

### Eligibility criteria

- Types of studies designs: randomized; non randomized; process evaluations studies and qualitative methods
- Types of population: UN agencies, local and international organizations and agencies including NGOs, governmental agencies and bodies
- Setting: individuals, groups, and communities during and after humanitarian crises. Examples of these crises include war, earthquake, and tsunami
- Types of interventions: mechanisms and models of coordination between organizations and agencies providing or financing health services. These could consist of one or more of the four categories of coordination mentioned above: information coordination, coordination through common representation, framework coordination and management/directive coordination
- Types of outcomes of measure:
  - Health outcomes of the affected population
  - Health outcomes of the host community
  - Access of the affected population to health services
  - Access of the host community to health services
  - Impact on health systems input

### Search strategy

We searched the following electronic databases: Medline, PubMed, EMBASE, Cochrane Central Register of Controlled Trials (CENTRAL), Cumulative Index to Nursing & Allied Health Literature (CINAHL), PsycINFO, WHO global Health Library (S1 Appendix). The search range was from the database date of inception till March 2014. Screening of the reference lists of included studies was also conducted to retrieve additional studies. S2 Appendix provides the free text terms and MeSH terms used to search the different electronic databases. We did not restrict the search to specific languages or dates.

In addition, we systematically searched in July 2014 the websites of the following organizations providing humanitarian interventions in the setting of crisis and conflicts: United Nations High Commissioner for Refugees (UNHCR), United Nations Office for the Coordination of Humanitarian Affairs (UN OCHA), International Organization for Migration (IOM), Centers for Disease Control and Prevention (CDC), Médecins sans frontières (MSF), International Medical Corps (IMC), Médecins du Monde (MDM), and United Nations Relief and Works Agency for Palestine Refugees (UNRWA). We used ‘coordination’, ‘cooperation’ and ‘collaboration’ as the search terms (S3 Appendix). We did not restrict the search to specific languages or dates.

### Selection process

Before starting the selection process, we conducted calibration exercises for all reviewers. We imported the results of the electronic databases search results into Endnote X7 and removed duplicates. We conducted the selection process of those results in two stages:

- Title and abstract screening: teams of two reviewers used the above eligibility criteria to screen titles and abstracts of identified citations in duplicate and independently for potential eligibility. We got the full text for citations judged as potentially eligible by at least one of the two reviewers.
- Full-text screening: a team of two reviewers used the same eligibility criteria to screen the full texts in duplicate and independently for eligibility. At this stage, the two reviewers compared results and resolved disagreement by discussion. When consensus could not be reached, a third reviewer made the final decision. We used standardized and pilot tested screening forms.
- As for the selection of the results of the website search, one reviewer went through the titles of the search hits. We then obtained the full text of those identified as potentially eligible, and two reviewers screened them in duplicate and independently. Then, they compared their results and resolved disagreement by discussion.

### Data abstraction process

Before starting the data abstraction process, we conducted calibration exercises to ensure the validity of the process. We used standardized and piloted data abstraction forms. Teams of two reviewers abstracted the data from eligible studies in duplicate and independently. Disagreements were resolved by discussion or with the help of a third reviewer when consensus could not be reached. Collected data included the following: type of study design, characteristics of the setting including the type of humanitarian crisis, date and location, population, the type of coordination and details about the mechanisms and models of coordination, types of health services provided or funded, funding, support and reported conflict of interest, outcomes assessed, statistical results and limitations of the study.

### Risk of bias assessment

We planned on assessing the risk of bias of the included studies using: the Cochrane Risk of Bias tool for randomized trials, a modified version of the Cochrane Risk of Bias tool for non-randomized studies, and the Critical Appraisal Skills Program (CASP) tool for qualitative studies.

### Data synthesis

We calculated the agreement between reviewers for the assessment of study eligibility at the full text screening stage using Fleiss' Kappa coefficient. We used the following values to judge the degree of agreement: 0.21–0.40 for fair agreement, 0.41–0.60 for moderate agreement, 0.61–0.80 for substantial agreement and 0.81–1.00 for almost perfect agreement.

For the quantitative analysis, we planned to:

- Calculate the relative risk (RR) for categorical data, for each study; and the mean difference (or, when appropriate, the standardized mean difference) for continuous data for each study.
- Pool the results across studies using a random-effects model, and test results for homogeneity across studies using the I^2^ test.
- Create inverted funnel plots of individual study results plotted against sample size in order to check for possible publication bias, if the number of identified studies allows.
- Report the results narratively.

For the qualitative analysis, we reported the results narratively and stratified them based on the type of emergency (e.g., war, earthquake, tsunami) and the type of intervention being considered (e.g., health clusters, health zones). We also reported the findings using the Joint Evaluation of Emergency Assistance to Rwanda Framework four categories: information coordination, coordination through common representation, framework coordination, and management/directive coordination [13].

We assessed the quality of evidence using the GRADE approach [14].

## Results

### Study selection

**Fig 1:**
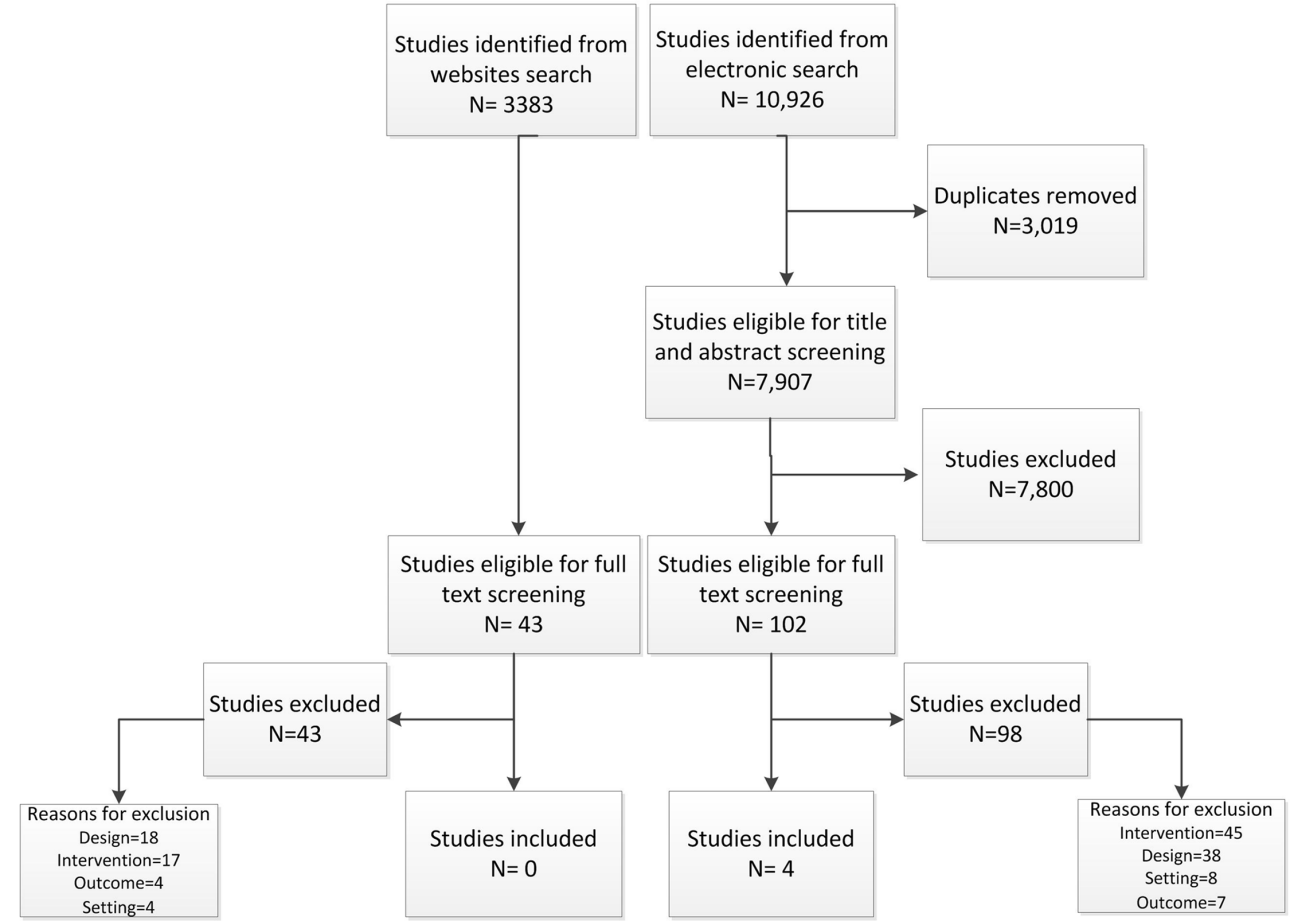
Selection Process Flowchart.

The study flow in Fig 1 summarizes the selection process. Out of 10,926 citations identified from electronic databases, four met the eligibility criteria [15–18]. At the full text screening, we excluded 98 articles for the following reasons: not intervention of interest (n = 45), not design of interest (n = 38), not setting of interest (n = 8), and not outcome of interest (n = 7). Table 1 provides the list of excluded studies with reasons for exclusion. The level of agreement between the two reviewers at the full text screening phase was good (Kappa = 0.614).

**Table 1:** Excluded studies with reasons for exclusion (databases search).

Study	Reason for Exclusion
Abebe, 2010 [24]	Not the appropriate study design
Ablah, 2007 [25]	Not the setting of interest
Ablah, 2010 [26]	Not the setting of interest
Abou Saleh, 2012 [27]	Not the setting of interest
Abrams, 2013 [28]	Not the intervention of interest
Ager, 2011 [29]	Not the appropriate study design
Altntas, 1999 [30]	Not the intervention of interest
Austin, 2008 [31]	Not the intervention of interest
Ayoya, 2013 [32]	Not the intervention of interest
Babcock, 2010 [33]	Not the intervention of interest
Baca, 2012 [34]	Not the appropriate study design, describes the mapping
Baingana, 2011 [35]	Not the appropriate study design
Barnes, 2012 [36]	Not the setting of interest
Bartschi, 2008 [37]	Not the appropriate study design
Bashir, 2003 [38]	Not the appropriate study design
Basikila, 1995 [39]	Not the intervention of interest
Benini, 1997 [40]	Not the appropriate study design
Bile, 2010 [41]	Not the appropriate study design
Bile, 2011 [42]	Not the appropriate study design
Bile, 2010 [43]	Not the appropriate study design
Bissel, 1994 [44]	Not the outcome of interest
Borton, 1996 [13]	Not the appropriate study design
Botoseneanu, 1996 [45]	Not the intervention of interest
Bremer, 2003 [46]	Not the intervention of interest
Burkle, 1995 [47]	Not the appropriate study design
Burkle, 2005 [48]	Not the intervention of interest
CDC, 1999 [49]	Not the appropriate study design
CDC&P, 1999 [50]	Not the intervention of interest
CDC, 2004 [51]	Not the intervention of interest
CDC, 2010 [52]	Not the appropriate study design, Descriptive
CDC, 2013 [53]	Not the setting of interest
Comfort, 2004 [54]	Not the appropriate study design, General-Theoretical
Curtis, 2008 [55]	Not the intervention of interest
Dar, 2011 [56]	Not the appropriate study design
Dhillon, 2012 [57]	Not the intervention of interest
Dolan, 2011 [58]	Not the intervention of interest—no coordination
Dominguez, 2012 [59]	Not the appropriate study design
Donev, 2002 [60]	Not the appropriate study design–descriptive
Dow, 1991 [61]	Not the intervention of interest–general
Drifmeyer, 2004 [62]	Not the setting of interest
Eloul, 2013 [21]	Not the appropriate study design
Emgushov, 2008 [63]	Not the appropriate study design
Fitzgerald, 2012	Not the appropriate study design
Gudi, 2010 [64]	Not the intervention of interest
Haar, 2012 [65]	Not the intervention of interest
Hector, 2011 [66]	Not the appropriate study design
Hossain, 2010 [67]	Not the intervention of interest
Henderson, 1983 [68]	Not the intervention of interest
Hunter, 2012 [69]	Not the intervention of interest
Jalali, 2002 [70]	Not the appropriate study design
James, 2012 [71]	Not the intervention of interest
Kang, 2012 [72]	Not the intervention of interest
Kapucu, 2011 [73]	Not the outcome of interest
Kirsch, 2012 [74]	Not the outcome of interest
Khankeh; 2011 [75]	Not the intervention of interest
Kirkpatrick, 2007 [76]	Not the intervention of interest
Kolaczinski, 2005 [77]	Not the appropriate study design–Descriptive
Kruke, 2012 [78]	Not the intervention of interest
Lanjouw, 1999 [79]	Not the intervention of interest
Lee, 2006 [80]	Not the intervention of interest
Libal, 2011 [81]	Not the appropriate study design
Liu, 2013 [82]	Not the outcome of interest
Maese, 2009 [83]	Not the appropriate study design
Markuland, 2010 [84]	Not the population of interest
Marshall, 2008 [85]	Not the setting of interest
Marshall, 2008 [86]	Not the intervention of interest
Martchenke, 1994 [87]	Not the intervention of interest
Matsumoto, 2013 [88]	Not the intervention of interest
McCann, 2011 [89]	Not the intervention of interest
McCabe, 2013 [90]	Not the intervention of interest
Male, 1996 [91]	Not the intervention of interest
Meynard, 2005 [92]	Not the intervention of interest
Miller, 2011 [93]	Not the intervention of interest
Montoya, 1987 [94]	Not the intervention of interest
Motamedi, 2009 [95]	Not the intervention of interest, no coordination
Myers, 2010 [96]	Not the intervention of interest; model for partnership not response
O’Connell, 2012 [97]	Not the intervention of interest; case studies
Oh, 2014 [19]	Not the outcome of interest
Ondos, 2007 [98]	Not the appropriate study design
Patel, 2013 [99]	Not the intervention of interest
Peak, 2006 [100]	Not the appropriate study design
Rechel, 2010 [101]	Not the intervention of interest
Rietjens, 2009 [102]	Not the appropriate study design
Shearer, 2007 [103]	Not the appropriate study design
Shen, 2012 [104]	Not the appropriate study design
Stephenson, 2005 [105]	Not the appropriate study design
Stumpenhorst, 2011 [106]	Not the appropriate study design
Subbarao, 2010 [107]	Not the appropriate study design
Tan, 2013 [108]	Not the appropriate study design
Tapia, 2012 [20]	Not the outcome of interest
Telford, 2004 [109]	Not the intervention of interest
Troy, 2008 [110]	Not the intervention of interest
Wiedrich, 2013 [111]	Not the intervention of interest; no coordination
Yanay, 2011 [112]	Not the appropriate study design
Yang, 2010 [113]	Not the outcome of interest
Zahner, 2005 [114]	Not the setting of interest
Zoraster, 2006 [115]	Not the appropriate study design
Zoraster, 2010 [116]	Not the appropriate study design

Out of the 3383 hits identified from the websites search, 43 reports were related to coordination in health setting. None of the 43 reports met the eligibility criteria for inclusion in our study. Exclusion reasons involved the following: not design of interest (n = 18), not intervention of interest (n = 17), not outcome of interest (n = 4) and not setting of interest (n = 4) as shown in Table 2.

**Table 2:** Excluded studies with reason of exclusion (websites search).

Study	Reason for Exclusion
AbouZahr, 2005 [117]	Not the setting of interest
CDC, 2011 [118]	Not the intervention of interest
Connolly, 2007 [119]	Not the intervention of interest
MDM, 2013 [120]	Not the intervention of interest
MDM, 2013 [121]	Not the intervention of interest
MDM, 2014 [122]	Not the intervention of interest
MDM, 2014 [123]	Not the outcome of interest
MDM, 2014 [124]	Not the intervention of interest
UNRWA, 2009 [125]	Not the appropriate study design
UNRWA, 2011 [126]	Not the appropriate study design
UNRWA, 2011 [127]	Not the appropriate study design
UNRWA, 2013 [128]	Not the appropriate study design
UNRWA, 2013 [129]	Not the appropriate study design
O’Heir, 2004 [130]	Not the intervention of interest
Robert, 2007 [131]	Not the appropriate study design
Reindorp, 2001 [132]	Not the intervention of interest
UNHCR, 2014 [133]	Not the appropriate study design
UNHCR, 2007 [134]	Not the appropriate study design
UNHCR, 2013 [135]	Not the appropriate study design
UNHCR, 1999 [136]	Not the intervention of interest
UNHCR, 2011 [137]	Not the appropriate study design
UNHCR, 2012 [138]	Not the appropriate study design
UNHCR, 2008 [139]	Not the intervention of interest
UNHCR, 2006 [140]	Not the intervention of interest
UNHCR, 2007 [141]	Not the intervention of interest
UNHCR, 2007 [142]	Not the intervention of interest
UNHCR, 2008 [143]	Not the appropriate study design
UNHCR, 2012 [144]	Not the appropriate study design
UN, 2000 [145]	Not the outcome of interest
UN, 2001 [146]	Not the outcome of interest
UN, 2008[147]	Not the appropriate study design
UNHCR, 2008 [148]	Not the design of interest
UNHCR, 2001 [149]	Not the intervention of interest
UN, 2013 [150]	Not the appropriate study design
UN, 2013 [151]	Not the appropriate study design
White, 2004 [152]	Not the outcome of interest
WHO, 2009 [153]	Not the intervention of interest
WHO, 2008 [154]	Not the setting of interest
WHO EMRO, 2003 [155]	Not the setting of interest
WHO EMRO, 2010 [156]	Not the setting of interest
WHO EMRO, 2010 [157]	Not the intervention of interest
WHO Indonesia fact sheets[158]	Not the design of interest

### Characteristics of included studies

S1 Table provides the characteristics of included studies in terms study method, setting and population, types of coordination, and outcomes.

#### Study methods

Out of the four included studies, two used mixed methods (both quantitative and qualitative) [15] [18], one used quantitative methods [16] and one used qualitative methods only [17]. Specific data collection methods included interviews (n = 3), field observations (n = 2), document analysis (n = 2) and content analysis of news reports (n = 1). None of the included studies employed a randomized controlled trial design. One study used network analysis to examine the coordination of relief efforts in humanitarian crisis [16]. This study used document analysis to construct the network. We did not conduct meta-analyses due to the lack of adequate quantitative data. Consequently, we reported the results narratively.

#### Setting and population

Three of the included studies took place in natural disaster settings: earthquake (n = 1) [15], flood (n = 1) [16] and cyclone (n = 1) [18]. Only one study examined coordination in post-conflict setting [17]. The main actors involved in providing assistance in humanitarian crisis setting and experimenting coordination included UN agencies (n = 3), local NGOs (n = 4), international NGOs (n = 3) and governmental agencies (n = 3).

#### Types of coordination

The included studies tackled two types of coordination between organizations and agencies providing humanitarian assistance: (1) information coordination in the form of the use of information and communication technologies [15]; and (2) management/directive coordination in the form of the humanitarian cluster approach [17], or coordination zones and cells [18]. One of the included studies did not clearly detail the mechanism of coordination employed [16]. Moore et al. described coordination as the flow of information and resources in a network, the number and strength of ties that an organization has with other organizations, joint activities and operations, communication and coordination meetings. None of the studies examined the two other types of coordination: the coordination through common representation and framework coordination [16].

#### Outcomes assessed

The included studies assessed the following outcomes:

- Access to health services measured as the association between coordination and number of beneficiaries [16] and as the number of health and medical care transactions [15].
- Impact on health system inputs assessed as the availability of medical services, products and human resources [18] and effective provision and quality of health services [17]. This assessment was based on perceptions of respondents and basic evaluation of data.

### Risk of bias assessment

Qualitative data: S2 Table shows the CASP assessment of the risk of bias of the three studies using qualitative methods. The three qualitative studies clearly stated the aim of the research and the value of research [15, 17, 18]. Two studies justified the way data is collected to address the research issue [15, 17]. Only one study took ethical issues into consideration [17] and one study considered the relationship between researcher and participants [17]. All studies reported and discussed their findings in an explicit way and in relation to other studies.

Quantitative data: We were not able to assess the risk of bias of the three studies using quantitative methods given the poor reporting of the methods and findings, and the descriptive nature of the studies [15, 16, 18].

In Table 1, we assessed the main limitations of each study. For example, Celik & Corbacioglo did not adjust for confounding that might affect the health and medical care function [15].

### Findings

We have organized the findings according to the type of coordination, categorized according to the Joint Evaluation framework [13]. As stated above, we identified data on information coordination and management/directive coordination but not on coordination through common representation or framework coordination.

#### Information coordination

Access to health services: two studies assessed this outcome and the results were as follows:

- Celik & Corbacioglo assessed the effect of information coordination particularly the use of information and communication technologies on disaster response performance measured using “emergency support functions and type of transactions”. They found an increase in the number of support functions and transactions for health and medical care, which improved from 8.36% before to 9.49% after (statistical significance not reported). The investigators assessed the effects of communication and coordination on 14 other functions, and found positive impact on four of them. Of note, the function mostly impacted was the search and rescue function [15].
- Moore et al. used the organization’s “centrality” to estimate its specific potential for aid coordination. They measured centrality through the number and strength of ties that an organization has with other organizations. Next, the investigators studied how centrality affected the number of NGO beneficiaries. They found statistically significant unadjusted associations between high centrality and the number of beneficiaries in areas of food and water and sanitation. This was in the context of emergency projects but not in the context of recovery projects. Moreover, while health is cited as one of the sectors of interest, the investigators did not report health specific results [16].

#### Management and directive coordination

Impact on health system inputs: two studies assessed this outcome and the results were as follows:

- Landegger et al. examined the strengths and weaknesses of the humanitarian cluster approach in relation to sexual and reproductive health including gender-based violence sub-cluster in Uganda following 20 years of civil war. The investigators reported that the humanitarian cluster approach improved the coordination among organizations working in sexual and reproductive health. They also reported that mapping within the cluster helped in improving the understanding of the availability of sexual and reproductive health services. The investigators additionally reported that the gender-based violence sub-cluster harmonized their strategy, reduced duplication and encouraged more effective provision of services. The cluster approach was found to enhance the quality of services through a common approach for providers’ training [17].
- Rahman & Bennish described the coordination efforts in Bangladesh following a cyclone in 1991. The health response was shown to be effective in terms of “a huge increase in drug availability and medical manpower”, and “much higher level of health services then they ever had before”. Although they reported “no significant increase in post-cyclone morbidity and mortality”, they did not provide data to support this conclusion [18].

Access to health services: The coordination intervention assessed by Moore et al. also had components of management coordination. Findings of this study are detailed above.

## Discussion

### Summary of findings

We identified very low quality evidence suggesting that information coordination between organizations, agencies and bodies providing health services in humanitarian crises settings may be effective in improving health systems inputs. There is additional very low quality evidence suggesting that management and directive coordination such as the cluster model may improve health system inputs in addition to access to health services. We identified no evidence of effectiveness for the two other categories of coordination, i.e., coordination through common representation and framework coordination.

### Research in the field

This review highlighted the limitations in the field of research in disaster and other humanitarian crisis settings. First, some of the included studies do not provide enough details about the coordination models being evaluated. These details could include the specific means by which the different organizations, agencies and bodies coordinated. For example, Rahman & Bennish provided detailed description of the model of coordination employed such as the agencies involved, the leading agencies and the establishment of coordination zones [18]. Such details are essential for organizations, agencies and bodies aiming to reproduce and implement these coordination mechanisms and models.

Second, not all coordination mechanisms and models have been assessed. As noted above, the included studies examined information and management coordination but none of them examined the other two forms of coordination, i.e., the coordination through common representation and framework coordination. This might be explained by the methodological challenges in assessing the two latter forms of coordination. The focus on information and management coordination maybe due to the fact that studies are assessing coordination in settings of rapid response to emergencies rather than response to chronic humanitarian situations.

A third limitation is the very low quality of the evidence provided by the available literature, weakening any inferences about effectiveness. None of the included studies used a controlled trial design as a way to minimize confounding or reported adjusting for confounding either. Similarly, the outcomes assessed in some of these studies were perceptions of respondents about effectiveness of coordination mechanisms, as opposed to the actual effectiveness.

Three of the four included studies examined coordination of relief efforts to sudden onset of emergencies such as earthquakes and natural disasters rather than in chronic humanitarian situations such as in the setting of armed conflicts and refugees. This makes the generalization of the findings to the latter situations more challenging.

Potential reasons for the limitations in the research work in this field include the acuteness and emergency nature of the subject, the lack of clear guidelines or standard on how to conduct and report studies in this field, and the scarcity of funding.

### Indirect evidence

We have identified, although not systematically, indirect evidence for our topic. This indirect evidence assesses the effectiveness of coordination between organizations, agencies and bodies providing health services other than health in humanitarian crises. One example is the study by Oh et al. [19]. The investigators focused on the brokerage role of international agencies to facilitate collaboration and coordination among the large number of agencies that participate and interact in a response network. Findings from a network analysis concluded that the use of international agencies as brokers, when the international organization took central position in the network and served as leading agency, can enhance the competencies of the overall emergency response system by serving as channeling agencies for critical resources and information.

In another example, Tapia et al. examined two humanitarian information coordination bodies: the Large International NGO Coordination (LINC) and the Organizational Change for Emergency Alliance (OCEA) [20]. The coordinating body has a focus, such as sharing information through technologies, and serves both to build a network and common capacity between organizations and to host several projects. Its objective is to find mechanisms for the multiple humanitarian organizations to coordinate around information technology and management. The study found that coordination bodies can increase the efficiency of the NGOs work particularly in using their technological powers and are promising strategies in building trust and relationships among organizations.

Closer to our topic, but not well developed in terms of research methods for providing empirical evidence, are publications describing coordination models for refugee health. For instance, the authors of a rich description of inter-agency coordination of mental health and psychosocial support for refugees and people displaced in Syria [21] reflect on the challenges and lessons learnt. They highlighted the incompatibility of an on-line coordination forum in a predominantly oral culture where electronic services are regularly disrupted, and, despite these difficulties, the need for sharing regularly updated information about staffing and activities.

### Strengths and limitations

To our knowledge this is the first systematic review of the effectiveness of coordination mechanisms and models between agencies and organizations providing health services in humanitarian crisis. The systematic review responds to priorities expressed by policymakers in the Eastern Mediterranean region and globally [7, 10, 11]. Furthermore, we conducted the review using standard, explicit, and rigorous methods [22]. Similarly, we followed recommended methods for reporting systematic reviews [23]. One of the major limitations of this systematic review, on the other hand, is that the findings are very limited in terms of quality and amount of evidence identified.

### Implication for policy and research

Although the identified evidence for the effectiveness of coordination mechanisms and models is limited, it still can help policymakers and stakeholders address coordination dysfunctions during humanitarian crisis including duplication of activities, inequitable distribution of aid, and poor access to essential health services. Stakeholder organizations may secure better access to essential and urgent healthcare needs of affected people by improving management and directive coordination. In the case of the Syrian refugee crisis in Lebanon, strengthening the stewardship function of governmental departments is critical. This, in addition to having a lead organization that is capable of playing a major role by coordinating and establishing effective partnerships with local and international agencies, donors, and academic institutions and conducting monitoring and evaluation.

Given the gaps and limitations identified, our systematic review findings can also inform researchers, and funders working or interested in the field. Researchers are encouraged to conduct more and better-designed studies examining the effectiveness of different coordination mechanisms and models between different organizations and agencies providing health services in humanitarian crises. In addition, process evaluation type of studies would help with better understanding the reasons for successes and failures in this field. Funders are encouraged to support the production of such studies. Research studies are needed in this field to better inform decision-making of different stakeholders working in providing and financing health services in humanitarian crisis. The evaluation research would benefit from better collaboration between academic researchers and organizations working in the field. Researchers are also encouraged to develop guidelines for conducting and reporting studies on coordination mechanisms in disaster settings given the complexity of evaluating effectiveness in such field. In the case of the Syrian refugee crisis in Lebanon, there is a need to identify research priorities on refugee health, shape research agendas and support studies to produce knowledge that can fill existing gaps. This would help develop and implement evidence-based interventions and provide policy guidance to improve coverage and access to essential health services.

Lastly, leading humanitarian organizations and bodies need to partner with research institutions, researchers and funders during crisis in order to identify research priorities and conduct context-specific research to inform policy and decision-making.

## Supporting Information

S1 Checklist (DOCX)

S1 Appendix Results of the searches of the electronic databases. (DOCX)

S2 Appendix Electronic databases search strategies. (DOCX)

S3 Appendix Websites Search. (DOCX)

S1 Table Characteristics of included studies. (DOCX)

S2 Table Assessment of methodological quality of qualitative studies using the CASP tool. (DOCX)

## References

[1] United Nations High Commissioner for Refugees (2014). Refugee Figures. Available: http://www.unhcr.org/pages/49c3646c1d.html

[2] United Nations High Commissioner for Refugees (2014). Internally Displaced People Figures. Available: http://www.unhcr.org/pages/49c3646c23.html

[3] United Nations High Commissioner for Refugees (2014) Syria Regional Refugee Response. Available: http://data.unhcr.org/syrianrefugees/regional.php

[4] United Nations High Commissioner for Refugees (1995) Refugee Health. Available: http://www.unhcr.org/cgi-bin/texis/vtx/search?page=search&docid=3ae68bf424&query=refugee%20health%201995

[5] Buse K, Walt G. (1996) Aid coordination for health sector reform: a conceptual framework for analysis and assessment Health Policy 38. 10.1016/0168-8510(96)00855-x 10162420

[6] Vayrynen R. (2001) Funding Dilemmas in Refugees Assistance: Political Interests and Institutional Reforms in UNHCR IMR 35: 143–167.

[7] The SPHERE Project (2014) Humanitarian Charter and Minimum Standards in Humanitarian Response. The SPHERE Project. 10.1111/j.0361-3666.2004.00245.x 20958782

[8] Rey F. (1999) The Complex Nature of Actors in Humanitarian Action and the Challenge of Coordination In Reflections on Humanitarian Action Pluto Press, London

[9] United Nations Office for the Coordination of Humanitarian Affairs UN OCHA (2014) Cluster Coordination.

[10] Evidence Aid Priority Setting Group (EAPSG) (2013) Prioritization of Themes and Research Questions for Health Outcomes in Natural Disasters, Humanitarian Crises or Other Major Healthcare Emergencies PLOS Currents Disasters 10.1371/currents.dis.c9c4f4db9887633409182d2864b20c31 PMC3805831 24162731

[11] Center for Systematic Review On Health Policy and Systems Research (SPARK) (2013) Priority Setting Meeting. Available: http://www.aub.edu.lb/spark/activities/Pages/prioritysetting.aspx

[12] Minear L CU, Crisp J, Mackinlay J, Weiss TG. UN coordination of the international humanitarian response to the Gulf Crisis 1990–1992 The Thomas J. Watson Institute for International Studies, Brown University, Providence, Rhode Island.

[13] Borton J (1996) An account of co-ordination mechanisms for humanitarian assistance during the international response to the 1994 crisis in Rwanda. Disasters 20: 305–323. 8991216 10.1111/j.1467-7717.1996.tb01046.x

[14] Guyatt G, Oxman AD, Akl EA, Kunz R, Vist G, Brozek J et al (2011) GRADE guidelines: 1. Introduction-GRADE evidence profiles and summary of findings tables. J Clin Epidemiol 64: 383–394. 10.1016/j.jclinepi.2010.04.026 21195583

[15] Celik S, Corbacioglu S (2010) Role of information in collective action in dynamic disaster environments. Disasters 34: 137–154. 10.1111/j.1467-7717.2009.01118.x 19682005

[16] Moore S, Daniel M, Eng E (2003) International NGOs and the role of network centrality in humanitarian aid operations: A case study of coordination during the 2000 Mozambique floods. Disasters 27: 305–318. 14725089 10.1111/j.0361-3666.2003.00235.x

[17] Landegger J, Hau M, Kaducu F, Sondorp E, Mayhew S, Roberts B (2011) Strengths and weaknesses of the humanitarian Cluster Approach in relation to sexual and reproductive health services in northern Uganda. International Health 3: 108–114. 10.1016/j.inhe.2011.03.005 24038183

[18] Rahman MO, Bennish M (1993) Health related response to natural disasters: The case of the Bangladesh cyclone of 1991. Social Science and Medicine 36: 903–914. 8480236 10.1016/0277-9536(93)90082-f

[19] Oh N, Okada A, Comfort LK (2014) Building Collaborative Emergency Management Systems in Northeast Asia: A Comparative Analysis of the Roles of International Agencies. Journal of Comparative Policy Analysis: Research and Practice.

[20] Tapia AH, Maldonado E, Tchouakeu L-MN, Maitland CF (2012) Coordinating humanitarian information: The problem of organizational and technical trajectories. Information Technology & People 25: 240–258.

[21] Eloul L, Quosh C, Ajlani R, Avetisyan N, Barakat M, Barakat L, et al (2013) Inter-agency coordination of mental health and psychosocial support for refugees and people displaced in Syria. Intervention: International Journal of Mental Health, Psychosocial Work & Counselling in Areas of Armed Conflict 11: 340–348.

[22] Higgins JPT GS (2011) Cochrane Handbook for Systematic Reviews of Interventions The Cochrane Collaboration

[23] Moher D LA, Tetzlaff J, Altman DG, The PRISMA Group (2009) Preferred Reporting Items for Systematic Reviews and Meta- Analyses: The PRISMA Statement. PLoS Med 6: e1000097 10.1371/journal.pmed.1000097 19621072 PMC2707599

[24] Abebe AM (2010) The aFrican Union Convention on internally displaced persons: Its codification background, scope, and enforcement challenges. Refugee Survey Quarterly 29: 28–57.

[25] Ablah E, Nickels D, Hodle A, Wolfe DJ, Orr S, Tenbrink J, et al (2007) "Public health investigation": a pilot, multi-county, electronic infectious disease exercise. American Journal of Infection Control 35: 382–386. 17660008 10.1016/j.ajic.2006.08.007

[26] Ablah E, Konda KS, Konda K, Melbourne M, Ingoglia JN, Gebbie KM. (2010) Emergency preparedness training and response among community health centers and local health departments: results from a multi-state survey. Journal of Community Health 35: 285–293. 10.1007/s10900-010-9236-7 20379843

[27] Abou-Saleh MT (2012) The World Federation for Mental Health: Building its constituency in the East Mediterranean Region for improving care and the lives of the mentally ill and their families. Arab Journal of Psychiatry 23: 178–184.

[28] Abrams JY, Copeland JR, Tauxe RV, Date KA, Belay ED, Mody RK, et al (2013) Real-time modelling used for outbreak management during a cholera epidemic, Haiti, 2010–2011. Epidemiology and Infection 141: 1276–1285. 10.1017/S0950268812001793 22935487 PMC9151838

[29] Ager A, Blake C, Stark L, Daniel T (2011) Child protection assessment in humanitarian emergencies: Case studies from Georgia, Gaza, Haiti and Yemen. Child Abuse and Neglect 35: 1045–1052. 10.1016/j.chiabu.2011.08.004 22099145

[30] Altntas KH, Delooz H (2004) The problems faced by three government disaster response teams of Ankara city during the Marmara earthquake—1999 response. Eur J Emerg Med 11: 95–101. 15028899 10.1097/00063110-200404000-00008

[31] Austin J, Guy S, Lee-Jones L, McGinn T, Schlecht J (2008) Reproductive Health: A Right for Refugees and Internally Displaced Persons. Reproductive Health Matters 16: 10–21. 10.1016/S0968-8080(08)31351-2 18513603

[32] Ayoya MA, Golden K, Ngnie-Teta I, Moreaux MD, Mamadoultaibou A, Koo L et al (2013) Protecting and improving breastfeeding practices during a major emergency: Lessons learnt from the baby tents in Haiti/Proteger et ameliorer les pratiques d'allaitement maternel au cours d'une situation d'urgence majeure: Les lecons tirees des tentes pour bebes en haiti. Bulletin of the World Health Organization 91: 612–617. 10.2471/BLT.12.113936 23940409 PMC3738309

[33] Babcock C, Baer C, Bayram JD, Chamberlain S, Chan JL, Galvin S, et al (2010) Chicago medical response to the 2010 earthquake in Haiti: translating academic collaboration into direct humanitarian response. Disaster medicine and public health preparedness 4: 169–173. 20526140 10.1001/dmphp.4.2.169

[34] Baca MJ, Fayyad K, Marini A, Weissbecker I (2012) The development of a comprehensive mapping service for mental health and psychosocial support in Jordan. Intervention: International Journal of Mental Health, Psychosocial Work & Counselling in Areas of Armed Conflict 10: 177–187.

[35] Baingana F, Mangen PO (2011) Scaling up of mental health and trauma support among war affected communities in northern Uganda: Lessons learned. Intervention: International Journal of Mental Health, Psychosocial Work & Counselling in Areas of Armed Conflict 9: 291–303.

[36] Barnes PA, Curtis AB, Hall-Downey L, Moonesinghe R (2012) A multistate examination of partnership activity among local public health systems using the national public health performance standards. Journal of Public Health Management & Practice 18: E14–23. 22836543 10.1097/PHH.0b013e31822ca424

[37] Bartschi E, Junker R, Lupi GA (2008) [Growing importance of the Coordinated Medical Services]. Ther Umsch 65: 36–41. 18399184 10.1024/0040-5930.65.1.36

[38] Bashir Z, Lafronza V, Fraser MR, Brown CK, Cope JR (2003) Local and state collaboration for effective preparedness planning. Journal of Public Health Management & Practice 9: 344–351. 15503597 10.1097/00124784-200309000-00003

[39] Basikila P, Male S, Lindgren J, Roberts L, Robinson D, Stettler N, et al (1995) Public health impact of Rwandan refugee crisis: What happened in Goma, Zaire, in July, 1994? Lancet 345: 339–344. 7646638

[40] Benini AA (1997) Uncertainty and information flows in humanitarian agencies. Disasters 21: 335–353. 9455006 10.1111/1467-7717.00066

[41] Bile KM, Lashari KA, Shadoul AF (2010) "Delivering as one" UN reform process to improve health partnerships and coordination: old challenges and encouraging lessons from Pakistan. Eastern Mediterranean Health Journal 16: S122–131. 21495598

[42] Bile KM, Hafeez A, Kazi GN, Southall D (2011) Protecting the right to health of internally displaced mothers and children: The imperative of inter-cluster coordination for translating best practices into effective participatory action/Proteger le droit a la sante des meres et des enfants deplaces a l'interieur de leur propre pays: Une coordination intergroupes est imperative pour traduire les meilleures pratiques en action participative efficace. Eastern Mediterranean Health Journal 17: 981–989. 22355953 10.26719/2011.17.12.981

[43] Bile KM, Shadoul AF, Raaijmakers H, Altaf S, Shabib K (2010) Learning through crisis: Development and implementation of a health cluster strategy for internally displaced persons. Apprendre grâce à la crise: Élaboration et mise en oeuvre d'une stratégie de groupe Santé en faveur des personnes déplacées 16: S82–90. 21495593

[44] Bissell RA, Pretto E, Angus DC, Shen B, Ruíz V, Ceciliano N, et al (1994) Post-preparedness medical disaster response in Costa Rica. Prehospital & Disaster Medicine 9: 96–106. 10155509 10.1017/s1049023x00040991

[45] Botoseneanu A, Wu H, Wasserman J, Jacobson PD (2011) Achieving public health legal preparedness: how dissonant views on public health law threaten emergency preparedness and response. Journal of Public Health 33: 361–368. 10.1093/pubmed/fdq092 21059686 PMC3159509

[46] Bremer R (2003) Policy development in disaster preparedness and management: lessons learned from the January 2001 earthquake in Gujarat, India. Prehospital and disaster medicine: the official journal of the National Association of EMS Physicians and the World Association for Emergency and Disaster Medicine in association with the Acute Care Foundation 18: 372–384. 10.1017/s1049023x00001345 15310051

[47] Burkle FM Jr, McGrady KA, Newett SL, Nelson JJ, Dworken JT, Lyerly WH Jr., et al (1995) Complex, humanitarian emergencies: III. Measures of effectiveness. Prehospital & Disaster Medicine 10: 48–56. 10155407 10.1017/s1049023x00041662

[48] Burkle FM. Integrating international responses to complex emergencies, unconventional war, and terrorism. Crit Care Med 33: S7–12. 15640683 10.1097/01.ccm.0000150955.49883.5c

[49] Centers for Disease Control (1999) Health status of and intervention for U.S.-bound Kosovar refugees—Fort Dix, New Jersey, May-July 1999. Mmwr Morbidity and mortality weekly report. 48: 729–732. 10503573

[50] Centers for Disease Control, Prevention (1999) Vaccination campaign for Kosovar Albanian refugee children—former Yugoslav Republic of Macedonia, April-May, 1999. MMWR—Morbidity & Mortality Weekly Report 48: 799–803. 10499784

[51] Centers for Disease Control, Prevention (2004) Emergency measles control activities—Darfur, Sudan, 2004. MMWR—Morbidity & Mortality Weekly Report 53: 897–899. 15457146

[52] Centers for Disease Control, Prevention (2010) Rapid establishment of an internally displaced persons disease surveillance system after an earthquake—Haiti, 2010. MMWR—Morbidity & Mortality Weekly Report 59: 939–945. 20689498

[53] Centers for Disease Contrl, Prevention (2013) CDC's Emergency Management Program activities—worldwide, 2003–2012. MMWR—Morbidity & Mortality Weekly Report 62: 709–713. 24005225 PMC4585622

[54] Comfort LK, Ko K, Zagorecki A (2004) Coordination in Rapidly Evolving Disaster Response Systems: The Role of Information. American Behavioral Scientist 48: 295–313.

[55] Curtis CA (2008) Communication and coordination among service and government organizations in New Orleans immediately following Hurricane Katrina. 1156 p.

[56] Dar OA, Khan MS, Murray V (2011) Conducting rapid health needs assessments in the cluster era: experience from the Pakistan flood. Prehospital and disaster medicine 26: 212–216. 10.1017/S1049023X11006261 22107774

[57] Dhillon P, Annunziata G (2012) The Haitian health Cluster experience: A comparative evaluation of the professional communication response to the 2010 earthquake and the subsequent cholera outbreak. PLoS Currents (SEP). 10.1371/5014b1b407653 PMC3470478 23074693

[58] Dolan B, Esson A, Grainger PP, Richardson S, Ardagh M (2011) Earthquake disaster response in christchurch, New Zealand. J Emerg Nurs 37: 506–509. 10.1016/j.jen.2011.06.009 21820725

[59] Dominguez PM, Victor IP (2012) Assessment of humanitarian actions in conflicts [Spanish]. Metas de Enfermería 15: 72–77.

[60] Donev D, Onceva S, Gligorov I (2002) Refugee crisis in Macedonia during the Kosovo conflict in 1999. Croatian Medical Journal 43: 184–189. 11885045

[61] Dow AA, Clark WE, Farmer JC, Nolan JP, Baskett PJ (1991) Disaster management. Organizations and academic perspective. Critical care clinics 7: 257–270. 2049639

[62] Drifmeyer J, Llewellyn C (2004) Toward more effective humanitarian assistance. Military Medicine 169: 161–168. 15080231 10.7205/milmed.169.3.161

[63] Emgushov O (2008) Coordinated care special needs shelter. Public Health Reports 123: 371–375. 19006979 10.1177/003335490812300317 PMC2289990

[64] Gudi A (2010) Effective knowledge integration in emergency response organizations. p.4354

[65] Haar RJ, Naderi S, Acerra JR, Mathias M, Alagappan K (2012) The livelihoods of Haitian health-care providers after the january 2010 earthquake: A pilot study of the economic and quality-of-life impact of emergency relief. International Journal of Emergency Medicine 5. 10.1186/1865-1380-5-13 PMC3327631 22385840

[66] Hector LJ (2011) A planning model for disaster relief agencies. 1082 p.

[67] Hossain L, Kuti M (2010) Disaster response preparedness coordination through social networks. Disasters 34: 755–786. 10.1111/j.1467-7717.2010.01168.x 20345465

[68] Henderson PL, Biellik RJ (1983) Comparative nutrition and health services for victims of drought and hostilities in the Ogaden: Somalia and Ethiopia, 1980–1981. International Journal of Health Services 13: 289–306. 6853004 10.2190/QGL7-V3U2-39D6-TELY

[69] Hunter JC, Crawley AW, Petrie M, Yang JE, Aragon TJ (2012) Local public health system response to the tsunami threat in coastal California following the Tohoku Earthquake. PLoS Currents (7 2012). 10.1371/4f7f57285b804 PMC3426142 22953236

[70] Jalali R (2002) Civil society and the state: Turkey after the earthquake. Disasters 26: 120–139. 12096846 10.1111/1467-7717.00196

[71] James T, Cubano M (2012) DOD and NGOs in Haiti—A successful partnership. World Medical and Health Policy 4.

[72] Shen J, Kang J, Shi Y, Li Y, Su L, Wu J, et al (2012) Lessons learned from the Wenchuan earthquake. Journal of Evidence-Based Medicine 5: 75–88. 10.1111/j.1756-5391.2012.01176.x 23557471

[73] Kapucu N (2011) Collaborative governance in international disasters: Nargis cyclone in Myanmar and Sichuan earthquake in China cases. International Journal of Emergency Management 8: 1–25.

[74] Kirsch T, Sauer L, Guha Sapir D (2012) Analysis of the international and US response to the Haiti earthquake: recommendations for change. Disaster Medicine & Public Health Preparedness 6: 200–208. 23077262 10.1001/dmp.2012.48

[75] Khankeh HR, Khorasani-Zavareh D, Johanson E, Mohammadi R, Ahmadi F, Mohammadi R. (2011) Disaster health-related challenges and requirements: a grounded theory study in Iran. Prehospital & Disaster Medicine 26: 151–157. 21929828 10.1017/S1049023X11006200

[76] Kirkpatrick DV, Bryan M (2007) Hurricane emergency planning by home health providers serving the poor. Journal of Health Care for the Poor & Underserved 18: 299–314. 17483559 10.1353/hpu.2007.0037

[77] Kolaczinski J (2005) Roll Back Malaria in the aftermath of complex emergencies: The example of Afghanistan. Tropical Medicine and International Health 10: 888–893. 16135196 10.1111/j.1365-3156.2005.01466.x

[78] Kruke BI, Olsen OE (2012) Knowledge creation and reliable decision-making in complex emergencies. Disasters 36: 212–232. 10.1111/j.1467-7717.2011.01255.x 21992233

[79] Lanjouw S, Macrae J, Zwi AB (1999) Rehabilitating health services in Cambodia: the challenge of coordination in chronic political emergencies. Health Policy Plan 14: 229–242. 10621240 10.1093/heapol/14.3.229

[80] Lee VJ, Low E (2006) Coordination and resource maximization during disaster relief efforts. Prehospital and disaster medicine: the official journal of the National Association of EMS Physicians and the World Association for Emergency and Disaster Medicine in association with the Acute Care Foundation 21: s8–12.

[81] Libal K, Harding S (2011) Humanitarian alliances: Local and international NGO partnerships and the iraqi refugee crisis. Journal of Immigrant and Refugee Studies 9: 162–178.

[82] Liu J, Wang S (2013) Building a new global search algorithm for managing the Chinese volunteer relief organisations. International Journal of Emergency Management 9: 187–204.

[83] Maese J (2009) Medical society's blueprint for a successful community response to emergency preparedness. Prehospital and disaster medicine: the official journal of the National Association of EMS Physicians and the World Association for Emergency and Disaster Medicine in association with the Acute Care Foundation 24: 73–75. 10.1017/s1049023x00006579 19557961

[84] Marklund LA, Graham AM, Morton PG, Hurst CG, Motola I, Robinson DW, et al (2010) Collaboration between civilian and military healthcare professionals: a better way for planning, preparing, and responding to all hazard domestic events. Prehospital & Disaster Medicine 25: 399–412. 21053185 10.1017/s1049023x00008451

[85] Marshall CS, Yamada S, Inada MK. Using problem-based learning for pandemic preparedness. Kaohsiung J Med Sci 24: S39–45. 18364286 10.1016/S1607-551X(08)70093-7 PMC7129008

[86] Marshall LW. International Disaster Response Law: an introduction. Am J Disaster Med 3: 181–184. 18666515

[87] Martchenke J, Rusteen J, Pointer JE (1995) Prehospital communications during the Loma Prieta earthquake… including commentary by Rottman SJ. Prehospital & Disaster Medicine 10: 225–231. 10161248 10.1017/s1049023x00042084

[88] Matsumoto H, Motomura T, Hara Y, Masuda Y, Mashiko K, Yokota H, et al (2013) Lessons learned from the aeromedical disaster relief activities following the Great East Japan earthquake. Prehospital and Disaster Medicine 28: 166–169. 10.1017/S1049023X12001835 23331849

[89] McCann DGC, Cordi HP (2011) Developing international standards for disaster preparedness and response: How do we get there? World Medical and Health Policy 3.

[90] McCabe OL, Perry C, Azur M, Taylor HG, Gwon H, Mosley A, et al (2013) Guided preparedness planning with lay communities: enhancing capacity of rural emergency response through a systems-based partnership. Prehospital & Disaster Medicine 28: 8–15. 23174414 10.1017/S1049023X12001483

[91] Male S (1996) Refugees: Do not forget the basics. World Health Statistics Quarterly 49: 221–224. 9170240

[92] Meynard JB, Nau A, Halbert E, Lelaidier M, Robin F, Todesco A. (2005) [Contribution of the French Armed Forces Health Services to relief in Indonesia following the tsunami on December 26, 2004]. Med Trop (Mars) 65: 113–116. 16038345

[93] Miller CW, McCurley MC (2011) Federal interagency communication strategies for addressing radiation emergencies and other public health crises. Health Physics 101: 559–561. 10.1097/HP.0b013e31822552d7 21979540

[94] Montoya D. Responding to disaster: Canada and the Mexico City earthquake. CMAJ 137: 68–70. 3594338 PMC1492410

[95] Motamedi MH, Saghafinia M, Bararani AH, Panahi F (2009) A reassessment and review of the Bam earthquake five years onward: what was done wrong? Prehospital and disaster medicine: the official journal of the National Association of EMS Physicians and the World Association for Emergency and Disaster Medicine in association with the Acute Care Foundation 24: 453–460. 10.1017/s1049023x00007317 20066651

[96] Myers L, Grant L (2010) The creation of regional partnerships for regional emergency planning. Journal of business continuity & emergency planning 4: 338–351. 21177220

[97] O'Connell R, Poudyal B, Streel E, Bahgat F, Tol W, Ventevogel P. (2012) Who is where, when, doing what: mapping services for mental health and psychosocial support in emergencies. Intervention: International Journal of Mental Health, Psychosocial Work & Counselling in Areas of Armed Conflict 10: 171–176.

[98] Ondos EJ (2008) An examination of the information technology used by Western Pennsylvania disaster managers for disaster readiness, response and relief efforts, and coordination. 4904 p.

[99] Patel A, Murakami N, Hamso M (2013) The evolving role of grass roots emergency medical response in the occupy wall street era: Hurricane sandy and the people's medical relief, Newyork city 2012. Journal of General Internal Medicine 28: S446–S447.

[100] Peake JB (2006) The Project HOPE and USNS mercy tsunami "experiment". Military Medicine 171: 27–29. 17447618 10.7205/milmed.171.1s.27

[101] Rechel B, Khodjamurodov G (2010) International involvement and national health governance: the basic benefit package in Tajikistan. Social Science & Medicine 70: 1928–1932. 20363064 10.1016/j.socscimed.2010.02.029

[102] Rietjens SJ, Verlaan K, Zaalberg TW, de Boer SJ (2009) Inter-organisational communication in civil-military cooperation during complex emergencies: a case study in Afghanistan. Disasters 33: 412–435. 10.1111/j.1467-7717.2008.01081.x 19178549

[103] Shearer D, Pickup F (2007) Still falling short: Protection and partnerships in the Lebanon emergency response. Disasters 31: 336–352. 18028157 10.1111/j.1467-7717.2007.01012.x

[104] Chen Z, Shen J, Kang JX, Shi YK, Li YP, Lin Su (2012) Emergency medical rescue after major earthquakes: Lessons from the Wenchuan earthquake. Chinese Journal of Evidence-Based Medicine 12: 383–392.

[105] Stephenson M Jr (2005) Making humanitarian relief networks more effective: Operational coordination, trust and sense making. Disasters 29: 337–350. 16277644 10.1111/j.0361-3666.2005.00296.x

[106] Stumpenhorst M, Stumpenhorst R, Razum O (2011) The un OCHA cluster approach: Gaps between theory and practice. Journal of Public Health 19: 587–592.

[107] Subbarao I, Wynia MK, Burkle FM Jr. (2010) The elephant in the room: collaboration and competition among relief organizations during high-profile disasters. J Clin Ethics 21: 328–334. 21313867

[108] Tan NT (2013) Policy and Collaboration for Social Recovery After Disaster. Journal of Social Work in Disability and Rehabilitation 12: 145–157. 10.1080/1536710X.2013.784606 23679810

[109] Telford J, Cosgrave J The international humanitarian system and the 2004 Indian Ocean earthquake and tsunamis. Disasters 31: 1–28. 17367371 10.1111/j.1467-7717.2007.00337.x

[110] Troy DA, Carson A, Vanderbeek J, Hutton A (2008) Enhancing community-based disaster preparedness with information technology. Disasters 32: 149–165. 10.1111/j.1467-7717.2007.01032.x 18217923 PMC2239245

[111] Wiedrich TW, Sickler JL, Vossler BL, Pickard SP (2013) Critical systems for public health management of floods, north dakota. Journal of Public Health Management & Practice 19: 259–265. 23348522 10.1097/PHH.0b013e3182641b39

[112] Yanay U, Benjamin S, Yamin HG (2011) Networking emergency teams in Jerusalem. Disasters 35: 183–199. 10.1111/j.1467-7717.2010.01199.x 20735459

[113] Yang Y (2010) The 9/21 earthquake in Taiwan: A local government disaster rescue system. Disasters 34: 112–136. 10.1111/j.1467-7717.2009.01117.x 19682006

[114] Zahner SJ (2005) Local public health system partnerships. Public Health Reports 120: 76–83. 10.1177/003335490512000113 PMC1497678 15736335

[115] Zoraster RM (2006) Barriers to disaster coordination: health sector coordination in Banda Aceh following the South Asia Tsunami. Prehospital and disaster medicine: the official journal of the National Association of EMS Physicians and the World Association for Emergency and Disaster Medicine in association with the Acute Care Foundation 21: s13–18.

[116] Zoraster RM (2010) Enhancing healthcare sector coordination through infrastructure and logistics support. American journal of disaster medicine 5: 215–219. 20879503

[117] AbouZahr C, Boerma T. (2005) Health information systems: the foundations of public health. Bulletin of the World Health Organization 83: 578–583. 16184276 PMC2626318

[118] Centers for Disease Control and Prevention (2011). Public Health Preparedness Capabilities: National Standards for State and Local Planning.

[119] Connolly MA, Gayer M, Ottmani S. (2007). Tuberculosis Care and Control in Refugee and Displaced Populations: An interagency field manual World Health Organization

[120] Médecins du Monde (2013). Syria: Today, Only Bombs Access to Civilian Populations.

[121] Médecins du Monde (2013). Syrie—Urgence Deux Ans Apres, Plus de 60,000 Victimes.

[122] Médecins du Monde (2014). HAITI: Reduction of There mortality and of There morbidity mother and child and fight against the cholera.

[123] Medecins du Monde (2014). NÉPAL: Renforcer l’accès et l’utilisation des services de santé sexuelle et reproductive.

[124] Medecins du Monde (2014). Syrie: Accès à la santé pour les populations déplacées et soutien aux médecins syriens.

[125] United Nations Relief and Works Agency for Palestine Refugees in the Near East (2009) Emergency Appeal for Northern Lebanon: Final Report

[126] United Nations Relief and Works Agency for Palestine Refugees (2011). The Annual Report of the Department of Health 2010.

[127] United Nations Relief and Works Agency for Palestine Refugees (2011). Field Implementation Plan 2010–2011.

[128] United Nations Relief and Works Agency for Palestine Refugees (2013). Health department annual report 2012.

[129] United Nations Relief and Works Agency for Palestine Refugees (2013) field implementation plan 2014–15: Syria.

[130] O’Heir J (2004) The Inter-agency Global Evaluation of Reproductive Health Services for Refugees and Internally Displaced Persons United Nations High Commissioner for Refugees.

[131] Robert WD, Phanwathanawong P. (2007) Strenghtening Protection Capacity Project Livelihoods Component: Phase Two International Labor Organization and United Nations High Commissioner for Refugees.

[132] Reindorp N, Wiles P. (2001) Humanitarian Coordination: Lessons from Recent Field Experience Office for the Coordination of Humanitarian Affairs (OCHA).

[133] United Nations High Commissioner for Refugees (2014) 2014 Syria Regional Response Plan Strategic Overview.

[134] United Nations High Commissioner for Refugees (2007) Country Operations Plan 2007: Syrian Arab Republic.

[135] United Nations High Commissioner for Refugees (2013) Syria Regional Response Plan: Egypt Response Plan.

[136] United Nations High Commissioner for Refugees (1999) Report on the Consultations Between UNHCR and Humanitarian and Human Rights NGOs in the Asia and Pacific Region on Strengthening Collaboration in Support of the International Refugee Protection System.

[137] United Nations High Commissioner for Refugees. (2011) Annual Report 2010 Public Health, Nutrition, HIV and WASH.

[138] United Nations High Commissioner for Refugees. (2012) Lebanon Update: Situation in North Lebanon.

[139] United Nations High Commissioner for Refugees (2008) Fighting hunger and malnutrition among UNHCR PoCs Summary of 2007 JAMs and Nutrition Surveys.

[140] United Nations High Commissioner for Refugees (2006) HIV/AIDS and Internally Displaced Persons in 8 Priority Countries.

[141] United Nations High Commissioner for Refugees (2007). Public Health Missions 2007.

[142] United Nations High Commissioner for Refugees (2007). Health, Nutrition and HIV/AIDS–New Strategies United Nations High Commissioner for Refugees—Executive Committee of High Commissioner Programme.

[143] United Nations High Commissioner for Refugees (2008). Annual Report: Public Health and HIV. United Nations High Commissioner for Refugees, Public Health and HIV Section, Division of Operational Services.

[144] United Nations High Commissioner for Refugees (2012). Update on coordination issues: strategic partenrships.

[145] United Nations—Economic and Social Council (2000) Strengthening of the coordination of emergency humanitarian assistance of the United Nations.

[146] United Nations—Economic and Social Council (2001) Strengthening the coordination of emergency humanitarian assistance of the United Nations.

[147] United Nations (2008) Report of the Commissioner-General of the United Nations Relief and Works Agency for Palestine Refugees in the Near East.

[148] United Nations High Commissioner for Refugees (2008) Annual Report: Urban Fact Sheet Public Health & HIV, Middle East and North Africa.

[149] United Nations High Commissioner for Refugees (2001) Reproductive Health Guide.

[150] United Nations (2013) Syria Regional Response Plan: regional Overview.

[151] United Nations (2013) Syria Regional Response Plan.

[152] White C (2004). Assessment of changes over time within agencies/institutions involved in Reproductive Health Services for refugees and internally displaced persons. The Inter-agency Global Evaluation of Reproductive Health Services for Refugees and Internally Displaced Persons.

[153] World Health Organization (2009) Protecting the health of vulnerable people from the umanitarian consequences of climate change and climate related disasters.

[154] World Health Organization—Regional Office of the Mediterranean (2008). Managing WHO Humanitarian Response in the Field World Health Organization.

[155] World Health Organization—Regional Office of the Mediterranean (2003) Country cooperation strategy for WHO and the Syrian Arab Republic 2003–2007.

[156] World Health Organization—Regional Office of the Mediterranean (2010). Country Cooperation Strategy for WHO and Afghanistan 2009–2013: Afghanistan

[157] World Health Organization—Regional Office of the Mediterranean (2010) Country Cooperation Strategy for WHO and Lebanon 2010–2015: Lebanon.

[158] World Health Organization Indonesia Fact Sheet. Health Sector Coordination

