# Supplementary material for: Effectiveness of Mechanisms and Models of Coordination between Organizations, Agencies and Bodies Providing or Financing Health Services in Humanitarian Crises: A Systematic Review
Source: PLoS One. 2015 Sep 2;10(9):e0137159. doi: 10.1371/journal.pone.0137159 (PMC4558048; doi:10.1371/journal.pone.0137159)
Supplement: S1 Appendix — (DOCX) [file pone.0137159.s002.docx]

# Appendix S1: Results of the searches of the electronic databases

| **Database** | **# of hits** | **Date of search** |
| --- | --- | --- |
| Medline (Ovid) | | |
|  | 2,006 | March 14, 2014 |
| PubMed | | |
|  | 769 | March 14, 2014 |
| WHO Global Health Library | | |
|  | 3,530 | March 2, 2014 |
| The Cochrane Library | | |
|  | 1 | March 5, 2014 |
| Cumulative Index to Nursing & Allied Health Literature (CINAHL) | | |
|  | 460 | March 14, 2014 |
| EMBASE (Ovid) | | |
|  | 2,523 | March 14, 2014 |
| Scopus | | |
|  | 580 | March 14, 2014 |
| PsychInfo | | |
|  | 1,057 (no books) | March 14, 2014 |
