# Supplementary material for: Effectiveness of Mechanisms and Models of Coordination between Organizations, Agencies and Bodies Providing or Financing Health Services in Humanitarian Crises: A Systematic Review
Source: PLoS One. 2015 Sep 2;10(9):e0137159. doi: 10.1371/journal.pone.0137159 (PMC4558048; doi:10.1371/journal.pone.0137159)
Supplement: S2 Appendix — (DOCX) [file pone.0137159.s003.docx]

# Appendix S2: Electronic databases search strategies

# Medline search strategy

Database: Ovid MEDLINE(R) <1946 to March Week 1 2014>

Search Strategy:

--------------------------------------------------------------------------------

1 exp Refugees/ (6558)

2 refugee*.ti,ab. (5394)

3 exp War/ (30340)

4 (war or wars).ti,ab. (25791)

5 disasters/ or exp disaster planning/ or exp emergency shelter/ or exp mass casualty incidents/ (23240)

6 (disaster* or tsunami* or earthquake* or volcan* or hurricane* or cyclone*).ti,ab. (20082)

7 (mass adj2 casualt*).ti,ab. (1355)

8 exp earthquakes/ or exp tsunamis/ or exp volcanic eruptions/ (2728)

9 ((conflict* adj3 (area* or zone* or setting* or region)) or (conflict-affected adj3 (area* or zone*or setting* or region)) or armed-conflict* or (armed adj3 conflict*) or (conflict adj3 ethnic) or post-conflict or postconflict or (military adj3 conflict) or "post conflict").ti,ab. (1770)

10 ((internal* adj2 (displace* or dis-place*)) or (forcibl* adj2 (displace* or dis-place*))).ti,ab. (422)

11 or/1-10 (84689)

12 (coordinat* or co-ordinat* or cooperat* or co-operat* or collaborat*).ti,ab. (297694)

13 exp cooperative behavior/ (28026)

14 international cooperation/ or medical missions, official/ (39498)

15 or/12-14 (346524)

16 organizations/ or exp charities/ or government/ or exp "united states dept. of health and human services"/ or exp local government/ or exp state government/ or exp government agencies/ or exp international agencies/ or organizations, nonprofit/ or foundations/ or voluntary health agencies/ (155048)

17 ((international or government* or non-government* or nongovernment* or nonprofit or non-profit or donor*) adj2 (organization* or organisation* or agenc* or bodies or foundation*)).ti,ab. (15316)

18 (united adj nation*).ti,ab. (3232)

19 (red adj cross).ti,ab. (2727)

20 ("world health" adj (organization or organisation)).ti,ab. (26650)

21 exp relief work/ or exp rescue work/ (4857)

22 ((relief or rescue) adj (work or effort*)).ti,ab. (395)

23 ((foreign or humanitarian) adj2 (aid or aids)).ti,ab. (497)

24 or/16-23 (193928)

25 15 and 24 (18828)

26 ((health adj3 (cluster* or inter-cluster or zone*)) or (cluster adj2 approach)).ti,ab. (952)

27 25 or 26 (19765)

28 11 and 27 (2006)

# EMBASE search strategy

# Database: Embase <1980 to 2014 Week 10>

Search Strategy:

--------------------------------------------------------------------------------

1 exp refugee/ (7321)

2 refugee*.ti,ab. (6074)

3 disaster/ or mass disaster/ or natural disaster/ or disaster planning/ (26347)

4 (disaster* or tsunami* or earthquake* or volcan* or hurricane* or cyclone*).ti,ab. (27440)

5 (mass adj2 casualt*).ti,ab. (1621)

6 exp earthquake/ (5038)

7 exp tsunami/ (1424)

8 exp volcano/ (2135)

9 war/ (24617)

10 (war or wars).ti,ab. (29491)

11 ((internal* adj2 (displace* or dis-place*)) or (forcibl* adj2 (displace* or dis-place*))).ti,ab. (548)

12 ((conflict* adj3 (area* or zone* or setting* or region)) or (conflict-affected adj3 (area* or zone*or setting* or region)) or armed-conflict* or (armed adj3 conflict*) or (conflict adj3 ethnic) or post-conflict or postconflict or (military adj3 conflict) or "post conflict").ti,ab. (2216)

13 or/1-12 (92212)

14 (coordinat* or co-ordinat* or cooperat* or co-operat* or collaborat*).ti,ab. (376241)

15 exp cooperation/ (39554)

16 coordination/ (3306)

17 exp international cooperation/ (157714)

18 or/14-17 (550057)

19 exp non profit organization/ (21069)

20 ((international or government* or non-government* or nongovernment* or nonprofit or non-profit or donor*) adj2 (organization* or organisation* or agenc* or bodies or foundation*)).ti,ab. (20577)

21 (united adj nation*).ti,ab. (4259)

22 (red adj cross).ti,ab. (3471)

23 ("world health" adj (organization or organisation)).ti,ab. (33513)

24 red cross/ (2731)

25 United Nations/ (6687)

26 world health organization/ (66809)

27 rescue work/ (573)

28 relief work/ (859)

29 ((relief or rescue) adj (work or effort*)).ti,ab. (466)

30 ((foreign or humanitarian) adj2 (aid or aids)).ti,ab. (454)

31 or/19-30 (136509)

32 18 and 31 (28322)

33 ((health adj3 (cluster* or inter-cluster or zone*)) or (cluster adj2 approach)).ti,ab. (1271)

34 32 or 33 (29576)

35 13 and 34 (2523)

# Scopus search strategy

# ((TITLE-ABS-KEY(refugee* OR disaster* OR earthquake* OR tsunami* OR hurricane* OR cyclone*or war OR wars OR conflict OR conflicts OR post-conflict)) OR (TITLE-ABS-KEY(internal* W/2 displace*)) OR (TITLE-ABS-KEY(forcibl* W/2 displace*)) OR (TITLE-ABS-KEY(mass W/2 casualt*))) AND (((TITLE-ABS-KEY(coordinat* OR co-ordinat* OR cooperat* OR co-operate* OR collaborat*)) AND (((TITLE-ABS-KEY((international OR government* OR non-government* OR nongovernment* OR nonprofit OR non-profit OR donor*) W/2 (organization* OR organisation* OR agenc* OR bodies OR foundation*))) OR (TITLE-ABS-KEY(united W/0 nation*)) OR (TITLE-ABS-KEY(red W/0 cross)) OR (TITLE-ABS-KEY("world health organization" OR "world health organisation"))) OR (TITLE-ABS-KEY(foreign W/1 aid)) OR (TITLE-ABS-KEY(humanitarian W/1 aid)) OR (TITLE-ABS-KEY(rescue W/1 (work OR effort*))) OR (TITLE-ABS-KEY(relief W/1 (work OR effort*))))) OR (TITLE-ABS-KEY(health W/2 cluster)))

# CINAHL search strategy

S12 S1 AND S11

S11 S7 OR S8 OR S9

S10 ((health N2 (cluster* or inter-cluster or zone*)) or (cluster N1 approach)) AND (S8 OR S9)

S9 (health N2 (cluster* or inter-cluster or zone*)) or (cluster N1 approach)

S8 international N2 cooperation

S7 S2 AND S6

S6 (S3 OR S4 OR S5)

S5 (rescue N2 work) or (relief N2 (work or effort*)) or (foreign N2 (aid or aids)) or (humanitarian N2 (aid or aids))

S4 "united nation*" or "red cross" or (world health N1 (organization or organization))

S3 (international or government* or non-government* or nongovernment* or nonprofit or non-profit or donor*) N2 (organization* or organisation* or agenc* or bodies or foundation*)

S2 coordinat* or co-ordinat* or cooperat* or co-operat* or collaborat*

S1 refugee* or (internal* N2 displace*) or (forcibl* N2 displace*) or war or wars or conflict or conflicts or disaster* or (mass N2 casualt*) or tsunami* or earthquake* or volcan* or hurricane* or cyclone*
