# Supplementary material for: Effectiveness of Mechanisms and Models of Coordination between Organizations, Agencies and Bodies Providing or Financing Health Services in Humanitarian Crises: A Systematic Review
Source: PLoS One. 2015 Sep 2;10(9):e0137159. doi: 10.1371/journal.pone.0137159 (PMC4558048; doi:10.1371/journal.pone.0137159)
Supplement: S3 Appendix — (DOCX) [file pone.0137159.s004.docx]

**Appendix S3: Websites Search**

| Website | Search terms used | # of hits | Date last searched |
| --- | --- | --- | --- |
| United Nations High Commissioner for Refugees (UNHCR)  <http://www.unhcr.org/cgi-bin/texis/vtx/home> | Coordination, collaboration or cooperation | 39 | July 5, 2014 |
| United Nations Office for the Coordination of Humanitarian Affairs (UN OCHA)  [www.unocha.org](http://www.unocha.org) | Coordination, collaboration or cooperation | 1616 | July 9, 2014 |
| International Organization for Migration (IOM)  [www.iom.int](http://www.iom.int) | Coordination, collaboration or cooperation | 192 | July 5, 2014 |
| World Health Organization (WHO)  [www.who.int](http://www.who.int) | Coordination, collaboration or cooperation | 732 | July 5, 2014 |
| Centers for Disease Control and Prevention (CDC)  [www.cdc.gov](http://www.cdc.gov) | Coordination, collaboration or cooperation | 257 | July 5, 2014 |
| Médecins sans frontiers (MSF)  [www.msf.org](http://www.msf.org) | Coordination, collaboration or cooperation | 133 | July 5, 2014 |
| International Medical Corps (IMC)  [www.internationalmedicalcorps.org](http://www.internationalmedicalcorps.org) | Coordination, collaboration or cooperation | 277 | July 5, 2014 |
| Médecins du Monde (MDM)  [www.medecinsdumonde.org](http://www.medecinsdumonde.org) | Coordination, collaboration or cooperation | 14 | July 5, 2014 |
| United Nations Relief and Works Agency for Palestine Refugees (UNRWA)  <http://www.unrwa.org/> | Coordination, collaboration or cooperation | 123 | July 9, 2014 |
