# Supplementary material for: Effectiveness of Mechanisms and Models of Coordination between Organizations, Agencies and Bodies Providing or Financing Health Services in Humanitarian Crises: A Systematic Review
Source: PLoS One. 2015 Sep 2;10(9):e0137159. doi: 10.1371/journal.pone.0137159 (PMC4558048; doi:10.1371/journal.pone.0137159)
Supplement: S1 Table — (DOCX) [file pone.0137159.s005.docx]

**Table S1:** Characteristics of included studies

| Celik 2010 | |
| --- | --- |
| Study setting | Earthquake in Marmara and Duzce, Turkey, 1999 |
| Population | Target of coordination: Emergency organizations, including private, public and non-profit organizations from different jurisdictional levels (local, national, provincial, and international) |
| Coordination | Type of coordination: information coordination  The use of information and communication technologies including the use of wireless communications and the availability of a shared knowledge and exchange of information between emergency organizations in disaster response led to “improved coordination”  Coordination was described as:   - Establishment of crisis management centers - Meetings - Organizational interactions - Resource-sharing activities |
| Design | Small-n exploratory case study used qualitative and quantitative methods.  Data sources:   - 58 semi-structured interviews with 39 key decision-makers and researchers - Content analyses of daily reports from Cumhuriyet, a daily Turkish newspaper - Field observations of disaster sites - Documents review |
| Outcome | Disaster response performance; measured using “emergency support functions and number of transactions” |
| Funding | Not reported |
| Limitations | The actual intervention was the use of information and communication technologies. It is not clear whether changes in disaster response performance are related directly to the use of technologies or to the improvement in coordination that resulted from the use of technologies. No adjustment for confounding. |
| Landegger, 2011 | |
| Setting | Post armed conflict in Uganda |
| Population | Governmental, non-governmental, United Nations, donor agencies, international and national NGOs and the World Health Organization (WHO) |
| Coordination | Type of coordination: management/directive coordination  Sexual and Reproductive Health and Gender-Based Violence (GBV) humanitarian Cluster Approach  The Cluster Approach aims to increase the effectiveness of humanitarian response by providing a platform for country governments, United Nations (UN) agencies and non-governmental organizations (NGO) to jointly improve capacity, organization, coordination, leadership and accountability within the different sectors of the humanitarian response both at the global and country level. |
| Design | Qualitative semi-structured interviews (face-to-face and telephone) |
| Outcome | Improved co-ordination of Sexual and Reproductive Health (SRH) services and stronger advocacy.  Cluster approach harmonized the strategy, reduced duplication, and encouraged more effective provision of GBV services. Respondents for the GBV interviews felt that the GBV sub-cluster had enhanced the quality of GBV services through a common approach for training GBV providers and monitoring tools. |
| Funding | Partially funded by the Reproductive Health Access, Information and Services in Emergencies Initiative (RAISE) |
| Limitations | The effectiveness of the cluster approach was only tested through the perception of respondents |
| Moore 2003 | |
| Setting | Flood in Mozambique in 2000 |
| Population | UN system agencies, bilateral national development agencies and international and local non-governmental  Organizations, LINK (Mozambican NGO) |
| Coordination | Type of coordination: information coordination and management/directive coordination  Organization’s centrality in the network was captured using three measures: (1) the flow of information and resources in a network (flow betweenness), (2) the number of ties that an organization has with other organizations and communication activity (degree centrality) and the strength of ties (eigenvector). Network ties were based on an organizations’ reporting of joint activities or operations with other organizations. |
| Design | - Field observations, interviews and documents analysis - Network analysis: the construction of three sets of social network matrices from which three centrality measures were generated - Quantitative hypothesis that tested the association between network centrality and beneficiary outcome numbers |
| Outcome | NGO average beneficiary numbers. |
| Funding | Not reported |
| Limitations | The analysis didn’t adjust for confounding such as the type of NGO, sector and province  Not enough details on the coordination mechanisms |
| Rahman 1993 | |
| Setting | Cyclone in Bangladesh in 1991 |
| Population | Government of Bangladesh (ministries of health, relief, and rehabilitation) and local administrative structures  Bangladesh armed forces, major donor agencies and organizations (UNICEF and WHO), local and international NGOs |
| Coordination | Type of coordination: management/directive coordination   - The coordination efforts included: - The establishment of the Emergency Relief Co-ordination Committee headed by the Prime Minister of Bangladesh to provide guidance to the Emergency Relief Management Committee which is headed by the Relief Minister in the Government of Bangladesh and consisting of key Secretaries of the Government. - The Emergency Relief Management Committee was responsible for coordinating and implementing all relief efforts. - The division of the affected area into two zones and the set-up of coordination cells at zonal, district and union level with representatives of the local civilian administration, the armed forces and relevant NGOs - The designation of NGOs with extensive local networks and experience as lead agencies in the distribution of health services such as the Association of Development Agencies of Bangladesh (ADAB) that facilitated NGO-NGO and NGO-government coordination. |
| Design | Evaluation of data (no further details provided) |
| Outcome | - The health response of Bangladesh cyclone was noted to be very effective. - Vast quantities of water purification tablets were supplied to the cyclone affected areas in response to an assumed wide-spread lack of drinking water. - Availability of medical services. - Rapid mobilization of health teams who provided acute medical care, and other primary health care services such as, distribution of oral rehydration solutions, antibiotics and other drugs and dissemination of health education messages. - Huge increase in drug availability and medical manpower following the cyclone. |
| Funding | Bangladesh office of UNICEF |
| Limitations | No clear methodology is mentioned.  It is not clear whether the outcome reported resulted from the coordination. |
