# Supplementary material for: Effectiveness of Mechanisms and Models of Coordination between Organizations, Agencies and Bodies Providing or Financing Health Services in Humanitarian Crises: A Systematic Review
Source: PLoS One. 2015 Sep 2;10(9):e0137159. doi: 10.1371/journal.pone.0137159 (PMC4558048; doi:10.1371/journal.pone.0137159)
Supplement: S2 Table — (DOCX) [file pone.0137159.s006.docx]

**Table S2:** Assessment of methodological quality of qualitative studies using the CASP tool

| Study Title | Year | Was there a clear statement of the aims of the research? | Is the qualitative methodology appropriate? | Was the research design appropriate to address the aims of the research? | Was the recruitment strategy appropriate to the aims of the research? | Was the data collected in a way that addressed the research issue? | Has the relationship between researcher and participants been adequately considered? | Have ethical issues been taken into consideration? | Was the data analysis sufficiently rigorous? | Is there a clear statement of findings? | How valuable is the research? |
| --- | --- | --- | --- | --- | --- | --- | --- | --- | --- | --- | --- |
| Celik | 2010 | Yes | No | Yes | Yes | Yes | No | No | Yes | Yes | yes |
| Rahman | 1993 | Yes | No | No | No | No | Can’t tell | Can’t tell | Can’t tell | Can’t tell | can't tell |
| Landegger | 2011 | Yes | Yes | Can’t tell | Yes | Yes | Yes | Yes | Yes | Yes | yes |
